# O5-5 Neighbourhood Walkability and Physical Activity: Moderating Role of a Physical Activity Interventions in Overweight and Obese Older Adults with Metabolic Syndrome

**DOI:** 10.1093/eurpub/ckac094.037

**Published:** 2022-08-29

**Authors:** Antoni Colom, Suzanne Mavoa, Maurici Ruiz, Julia Wärnberg, Josep Muncunill, Jadwiga Konieczna, Francisco Javier Barón-López, Guillem Vich, Montserrat Fitó, Jordi Salas-Salvadó, Dora Romaguera

**Affiliations:** 1 Research Group on Nutritional Epidemiology & Cardiovascular Physiopathology, Health Research Institute of the Balearic Islands (IdISBa), Palma de Mallorca, Spain; 2 CIBER Fisiopatología de la Obesidad y Nutrición (CIBEROBN), Instituto de Salud Carlos III, Madrid, Spain; 3 Mary MacKillop Institute for Health Research, Australian Catholic University, Melbourne, Australia; 4 Melbourne School of Population and Global Health, University of Melbourne, Melbourne, Australia; 5 Servicio de SIG y Teledetección, Vicerectorat d'Innovació i Transferència, Universitat de les Illes Balears, Palma de Mallorca, Spain; 6 Departamento de Enfermería, Facultad de Ciencias de la Salud, Universidad de Málaga, Málaga, Spain; 7 Genomics and Bioinformatics Platform', Health Research Institute of the Balearic Islands (IdISBa), Palma de Mallorca, Spain; 8 Departamento de Salud Pública, Facultad de Medicina, Universidad de Málaga, Malaga, Spain; 9 Geography Department, Autonomous University of Barcelona, Barcelona, Spain; 10 Unit of Cardiovascular Risk and Nutrition, Institut Hospital del Mar de Investigaciones Médicas Municipal d'Investigació Mèdica (IMIM), Barcelona, Spain; 11 Department of Biochemistry and Biotechnology, Human Nutrition Unit, Rovira i Virgili University, Reus, Spain

**Keywords:** longitudinal study, physical activity intervention, built environment, walkability index, older adults

## Abstract

**Background:**

While urban built environments might promote active ageing, an infrequently studied question is how the neighbourhood walkability modulates physical activity changes during a physical activity intervention program in older adults. We assessed the influence of objectively assessed neighbourhood walkability on the change in physical activity during the intervention program used in the ongoing PREvención con DIeta MEDiterránea (PREDIMED)-Plus trial. PREDIMED-Plus is a parallel-group, randomized trial which tested the effect of an intensive lifestyle intervention on cardiovascular disease prevention, in overweight and obese participants with the metabolic syndrome.

**Method:**

The present study involved 228 PREDIMED-Plus senior participants aged between 55 to 75, recruited in Palma de Mallorca (Spain). Overweight/obese older adults with metabolic syndrome were randomized to an intensive weight-loss lifestyle intervention or a control group (106 intervention group and 122 control group). A home neighborhood environment walkability index (residential density, land use mix, intersections density) was calculated using geographic information systems (1km sausage-network buffer). Physical activity was assessed using accelerometer for seven days, and a REGICOR validated physical activity questionnaire, at baseline and 2 follow-up visits (six-months and one-year later). Generalized Additive Mixed Models (GAMMs) were fitted to estimate the association between the neighbourhood walkability index and changes in physical activity during follow-up.

**Results:**

Higher neighbourhood walkability (1 z-score increment) was associated with moderate-to-vigorous accelerometer assessed physical activity duration, (ß = 3,44; 95% CI = 0.52;6.36 minutes per day). When analyses were stratified by intervention arm, the association was only observed in the intervention group (ß = 6.357; 95% CI = 2.07;10.64 minutes per day) (p for interaction = 0.055). There were no statistically significant associations between neighbourhood walkability and self-reported physical activity nor brisk walking duration.

**Conclusions:**

The results indicate that the walkability of the neighbourhood could support a physical activity intervention, helping to maintain or increase older adults' objectively measured physical activity. This research may modify evidence on whether environmental factors modify habits acquisition during physical activity intervention programs.

